# Effect of socioeconomic status and healthcare provider on post-transplantation care in Malaysia: A multi-centre survey of kidney transplant recipients

**DOI:** 10.1371/journal.pone.0284607

**Published:** 2023-04-19

**Authors:** Peter Gan Kim Soon, Sanjay Rampal, Soo Kun Lim, Tin Tin Su

**Affiliations:** 1 Planning Division, Ministry of Health Malaysia, Ipoh, Malaysia; 2 Department of Social and Preventive Medicine, Faculty of Medicine, University of Malaya, Lumpur, Malaysia; 3 Department of Medicine, Faculty of Medicine, University of Malaya, Lumpur, Malaysia; 4 South East Asia Community Observatory (SEACO), Jeffery Cheah School of Medicine and Health Sciences, Monash University Malaysia, Subang Jaya, Malaysia; Universiti Teknologi Malaysia, MALAYSIA

## Abstract

**Introduction:**

As the rate of end-stage kidney disease rises, there is an urgent need to consider the catastrophic health expenditure of post-transplantation care. Even a small amount of out-of-pocket payment for healthcare can negatively affect households’ financial security. This study aims to determine the association between socioeconomic status and the prevalence of catastrophic health expenditure in post-transplantation care.

**Method:**

A multi-centre cross-sectional survey was conducted in person among 409 kidney transplant recipients in six public hospitals in the Klang Valley, Malaysia. Catastrophic health expenditure is considered at 10% out-of-pocket payment from household income used for healthcare expenditure. The association of socioeconomic status with catastrophic health expenditure is determined via multiple logistic regression analysis.

**Results:**

93 kidney transplant recipients (23.6%) incurred catastrophic health expenditures. Kidney transplant recipients in the Middle 40% (RM 4360 to RM 9619 or USD 1085.39 –USD 2394.57) and Bottom 40% (<RM 4,360 or < USD 1085.39) income groups experienced catastrophic health expenditure compared to the Top 20% (>RM 9619 or > USD 2394.57) income group. Kidney transplant recipients in the Bottom 40% and Middle 40% income groups were more susceptible to catastrophic health expenditure at 2.8 times and 3.1 times compared to higher-income groups, even under the care of the Ministry of Health.

**Conclusion:**

Universal health coverage in Malaysia cannot address the burden of out-of-pocket healthcare expenditure on low-income Kidney transplant recipients for long-term post-transplantation care. Policymakers must reexamine the healthcare system to protect vulnerable households from catastrophic health expenditures.

## Introduction

It is universally acknowledged that affordable, accessible and quality healthcare provision is a fundamental right. Nevertheless, households still experience financial risk for seeking healthcare, widely known as catastrophic health expenditure (CHE) across the globe [1, 2]. Xu et al. demonstrated that the incidence of CHE depends on the country’s healthcare system and its capacity to provide a safety net for the vulnerable population [3]. High-income countries protect their population from CHE with appropriate risk pooling strategies, including health insurance coverage and a tax-based financed healthcare system [3, 4]. In contrast, low- and middle-income countries (LMIC) lack effective risk-pooling mechanisms in the healthcare financing systems, which could lead to CHE [3, 5]. However, it is not necessary to have high healthcare costs to be considered financially catastrophic. In LMIC, even a small amount paid out-of-pocket (OOP) for healthcare can have impoverishing effects on the economic security of the households to maintain their sustenance needs [3], especially those at the lower socioeconomic gradient [6–9].

Malaysia was recognised to have achieved universal health coverage (UHC) in 1980 by investing and prioritising the healthcare system, especially for rural communities [10]. Malaysia’s healthcare system is a dichotomy of near-free tax-based, government-run and private hospitals, predominantly financed by OOP or private health insurance [11]. The government-run hospitals are principally under the Ministry of Health (MOH), with a few university hospitals under the Ministry of Education (MOE) with nephrology services. Most of the government’s funding for renal replacement therapy is directed towards dialysis rather than transplant, which leads to inadequate healthcare resources [12]. Although kidney transplant recipients (KTRs) attending public hospitals under the MOH are provided with near-free service and medications; however, those under the care of the MOE_ hospitals are required to pay OOP for anti-rejection medications and other medications.

Coincident with the rising interest in equitable healthcare, many studies in high-income countries have examined the socioeconomic disparities in kidney transplantation [13, 14], highlighting the association between socioeconomic inequality access and the outcome of kidney transplantation. On the contrary, little is known about the association of socioeconomic status (SES) of KTRs on CHE of post-transplantation care in LMIC countries, including Malaysia. With the increasing burden of non-communicable diseases such as end-stage kidney disease (ESKD), OOP payment in Malaysia has proportionally increased to 38% [15]. Bavanandan et al. conducted a study in Malaysia that comprehensively investigated the cost and utility of kidney transplantation on the healthcare system from providers’ perspectives [16]. However, they did not consider the expenditure on the post-transplantation care of KTRs.

Most studies reported on the cost savings for kidney transplantation from a health provider/healthcare system perspective [17–19], but only a few from a patient perspective. Despite kidney transplantation being the preferred renal replacement therapy (RRT) for suitable ESKD patients [20], the 24^th^ Report of the Malaysian Dialysis and Transplant Registry only registered 82 ESKD patients who received renal transplantation compared to 7663 ESKD patients who received dialysis in 2016 [21]. Kidney transplantation aims to improve the quality of life of ESKD patients in addition to prolonging their life span [22, 23]. If the healthcare system cannot provide adequate protection from CHE, it may offset the benefits of kidney transplantation. Therefore, this study aims to determine the effect of socioeconomic status and healthcare providers on post-transplantation care in Malaysia.

## Method

A cross-sectional study was conducted between February and June 2018 at six public hospitals providing post-transplantation care in the Klang Valley. According to the National Renal Registry, these hospitals were selected because they provide post-transplantation care to nearly half of all the KTRs in Malaysia. All four transplant centres in Malaysia are located in the Klang Valley. The region is the most industrialised and densely populated region in Malaysia.

Primary data was obtained from a multi-centre cross-sectional study conducted between February and June 2018 at six public hospitals providing post-transplantation care in the Klang Valley; Kuala Lumpur Hospital (n = 168), Selayang Hospital (n = 79), Serdang Hospital (n = 20), Tengku Ampuan Rahimah Hospital (n = 20), Universiti Malaya Medical Centre (n = 85) and Universiti Kebangsaan Malaysia Medical Centre (n = 37). Therefore, the study was exploratory, using a universal sampling approach to recruit the study participants.

A sample size of 350 participants was required by assuming a proportion of 50% [24], with an 80% power to detect this difference at the 5% significance level after considering a non-response rate of 30% [25]. During the data collection period, the Malaysian Dialysis and Transplant Registry recorded 885 KTRs under the care of the six hospitals mentioned above, constituting almost half of the 1814 KTRs in the country [26].

To be eligible for this study, KTRs need to be of Malaysian nationality above 18 years old. Participants were required to understand and self-administer the survey in either Malay, English or Chinese. KTRs who were diagnosed with graft failure and made to undergo dialysis were excluded. KTRs diagnosed with acute organ rejection and transplanted less than six months from recruitment were also excluded because the clinical follow-ups would be more regular and inflate the medical expenditure.

A self-administered questionnaire was developed from established literature on CHE [3, 27, 28]. Experts in the field validated the survey tool—each item achieved a content validation index between 0.78 and 1.00 and a Kappa value between 0.75 and 1.00. The questionnaire was translated into Malay and Chinese. Face validation of the final questionnaire was piloted at a tertiary hospital providing post-transplantation care. Personal information such as sociodemographic information of KTRs and their health status (illness in previous four weeks and presence of comorbid conditions), health service utilisation, and household information like household income, expenditure and OOP healthcare expenditure were collected. The first author performed the face-to-face interviews using the standardised questionnaire conducted within the premises of the outpatient clinic of the hospitals.

CHE is calculated as the total direct medical expenditure over total household income in the four weeks preceding the survey [29, 30]. A threshold of 10% of household income on post-renal transplantation care was used, which is adopted by the Sustainable Development Goals to monitor UHC protection [1]. Direct medical expenses were used to estimate the OOP and include the cost of inpatient and outpatient care for medicines, admission or registration fees, physician/consultation fees, and diagnostic test fees. Payments for transportation, lodging, and food during follow-ups were excluded. The direct health expenditure (X) for KTRs’ post-transplantation care can be measured as follows [31].

$$X=\frac{M_{exp}}{H_{in}}$$

Where, *X* is the direct health expenditure for post-transplantation care, *M*_*exp*_ is the average direct medical expenditure for post-transplantation, and *H*_*in*_ is the average total household income of the KTRs. The socioeconomic status gradient was assessed using educational attainment, household income and employment status of the KTRs. Education attainment levels were categorised as no formal education or primary education, secondary education, and tertiary education. Household income is classified as Bottom 40% (<RM 4,360 or < USD 1085.39), Middle 40% (RM 4360 to RM 9619 or USD 1085.39 –USD 2394.57), and Top 20% (>RM 9619 or > USD 2394.57) [32]. The currency exchange of the Malaysian Ringgit to the US Dollar is based on Bank Negara’s foreign exchange rate for 2020 [33]. Employment status was categorised as employed, outside workforce, and unemployed.

Baseline characteristics for sociodemographic, medical and financial characteristics of the KTRs were tabulated as mean ± SD for continuous variables and frequencies and relative percentages [34] for categorical variables (Tables 1 and 2). Differences between proportions were tested using Pearson’s Chi-Square test. Differences between the two means were tested using the independent-sample T-Test. The difference between three or more means was tested using a One-ANOVA test.

**Table 1:** General characteristics of kidney transplant recipients by household income.

Characteristics	Total (N)	Frequency, n (%)
**Age, mean ± SD**	398	47.2 ± 14.1
**Gender**	398	
Male		223 (56.0)
Female		175 (44.0)
**Ethnicity**	398	
Malay		127 (31.9)
Chinese		220 (55.3)
Indian		42 (10.6)
Others		9 (2.3)
**Educational Attainment**	398	
No Formal Education / Primary		59 (14.8)
Secondary		158 (39.7)
Tertiary		181 (45.5)
**Employment Status**	398	
Unemployed		43 (10.8)
Outside Workforce		104 (26.1)
Employed		251 (63.1)
**Marital Status**	398	
Unmarried		113 (28.4)
Married		263 (66.1)
Others		22 (5.6)
**Geographical Location**	397	
Klang Valley		112 (28.2)
Others		285 (71.8)
**Post-transplantation care expenditure**		
**Out-of-pocket Payment** ^**1**^	398	
Current income		245 (61.6)
Savings		139 (34.9)
Loan		39 (9.8)
Selling Assets		18 (4.5)
Reduce Household Spending		78 (19.6)
**Public Financing** ^**1**^	398	313 (78.6)
**Private Financing** ^**1**^	398	68 (17.1)
**Perceived financial burden on health expenditure**	397	
A Little		208 (52.4)
Moderate		138 (34.8)
Extreme		51 (12.8)

SD, standard deviation; IQR, Interquartile range

^1^ Out-of-pocket payment, public financing and private financing are questions with multiple choice for kidney transplant recipients to select

^#^ Bottom 40% (<RM 4,360 or < USD 1085.39), Middle 40% (RM 4360 to RM 9619 or USD 1085.39 –USD 2394.57), and Top 20% (>RM 9619 or > USD 2394.57)

* Outside workforce consists of homemakers, students and retireesConversion rate, RM 1 = USD 0.249 [33]

**Table 2:** Medical characteristics of kidney transplant recipients by household income.

Characteristics	Total (N)	Frequency, n (%)
**Pre-Transplantation**		
** Donor type**	397	
Living		251 (63.1)
Deceased		146 (36.7)
** Transplant Centre**	398	
Local		242 (60.8)
Overseas		156 (39.2)
**Duration of Dialysis (months), Median (IQR)**	398	12.0 (4.25, 36.0)
**Post-Transplantation**		
** Follow-up Compliance**	398	336 (84.4)
** Medication Compliance**	398	388 (97.5)
**Duration Since Transplant (months),** **Median (IQR)**	398	108.0 (48.0, 168.0)
** Healthcare Provider**	398	
Ministry of Health		279 (70.1)
Ministry of Education		119 (29.9)

^**1**^ The comorbidities and perceived preparedness are questions with multiple choice for kidney transplant recipients to select yes or no

^#^ Bottom 40% (<RM 4,360 or < USD 1085.39), Middle 40% (RM 4360 to RM 9619 or USD 1085.39 –USD 2394.57), and Top 20% (>RM 9619 or > USD 2394.57)

IQR, interquartile range

Conversion rate, RM 1 = USD 0.249 [33]

The CHE was estimated using Greene’s logit equation [35]. While the model goodness-of-fit was measured using Hosmer–Lemeshow test [36]. Where, y is the presence of catastrophic health expenditure (Yes = 1, Otherwise = 0), x_j_ is a set of predetermined variables β, a set of parameters to be estimated.

$$\mathrm{Pr}[y=1]=\frac{\exp\left(x_{j}\beta \right)}{1+\exp\left(x_{j}\beta \right)}$$

The prevalence of CHE was estimated using the survey command in Stata (syy linearised) to account for sampling the participants from six different hospitals (Table 3). Taylor-linearised variance estimation was utilised to obtain the standard error of the variance. Differences in prevalence were tested using the F statistics from the Rao-Scot correction of the Chi-Square test.

**Table 3:** Prevalence of catastrophic health expenditure by levels of socioeconomic status and healthcare provider.

Characteristics	Total KTRs (N)	Catastrophic Health Expenditure, % (SE)	P-Value
**Total KTRs**	**396**		**N/A**
**Overall Household Income** ^**#**^			**0.005**
Bottom 40%	150	25.3% (0.03)	
Middle 40%	147	29.7% (0.04)	
Top 20%	99	12.1% (0.03)	
**Educational Attainment**			0.357
No Education / Primary	58	29.3% (0.06)	
Secondary	158	24.7% (0.03)	
Tertiary	180	20.6% (0.03)	
**Employment Status**			0.942
Unemployed	43	11.8% (0.07)	
Outside workforce	104	25.8% (0.04)	
Employed	249	62.4% (0.03)	
**Healthcare Provider**			**<0.001**
Ministry of Health	277	12.3% (0.02)	
Ministry of Education	119	49.6% (0.02)	

^#^ Bottom 40% (<RM 4,360 or < USD 1085.39), Middle 40% (RM 4360 to RM 9619 or USD 1085.39 –USD 2394.57), and Top 20% (>RM 9619 or > USD 2394.57)

SE, standard error

Conversion rate, RM 1 = USD 0.249 [33]

Multivariable mixed effects logistic regression was used to model the prevalent odds of CHE (Tables 4 and 5). This model accounted for data clustering by hospitals and adjusted the associations between socioeconomic gradient indicators (educational attainment, household income, and type of healthcare provider) and CHE for confounding [37]. The selection of confounders (age, gender, ethnicity, and geographical location) was a priori and based on epidemiological plausibility [38, 39]. There was no evidence of collinearity among the variables in the adjusted model (VIF<4). Data management and analysis were performed using International Business Machines (IBM) Statistical Package for the Social Sciences (SPSS version 22) and Stata version 17.

**Table 4:** Association between levels of socioeconomic status, healthcare provider and prevalence of catastrophic health expenditure.

Characteristics	Crude Odds Ratio (95% CI)	P-Value	Adjusted Odds Ratio (95% CI)	P-Value
**Household Income** ^**1a**^^,^ ^**#**^				
Bottom 40%	2.5 (1.2, 5.0)	**0.013**	2.8 (1.4, 6.0)	**0.002**
Middle 40%	3.1 (1.5, 6.2)	**0.002**	3.1 (1.5, 6.3)	**0.005**
Top 20%	1 (reference)		1 (reference)	
**Education Attainment** ^**1b**^				
No / Primary	1.6 (0.8, 3.1)	0.365	1.5 (0.7,3.2)	0.243
Secondary	1.3 (0.8, 2.1)	0.168	1.1 (0.6,1.8)	0.813
Tertiary	1 (reference)		1 (reference)	
**Employment Status** ^**1c**^				
Unemployed	0.7 (0.3, 1.7)	0.429	0.6 (0.2, 1.4)	0.225
Outside workforce	0.9 (0.5, 1.6)	0.675	0.9 (0.5, 1.7)	0.704
Employed	1 (reference)		1 (reference)	
**Healthcare Provider** ^**1d**^				
Ministry of Health	1 (reference)		1 (reference)	
Ministry of Education	7.0 (4.2, 11.7)	**<0.001**	8.6 (5.0, 14.9)	**<0.001**

^1a^ Adjusted for age, age^2^, gender, ethnicity, geographical location, employment status and household income

^1b^ Adjusted for age, age^2^, gender, ethnicity, and geographical location

^1c^ Adjusted for age, age^2^, gender, ethnicity, geographical location and educational attainment

^1d^ Adjusted for household income and education attainment

^#^ Household income classified as Bottom 40%, <RM4360 (< USD 1042.04) Middle 40%, RM4360–RM9619 (USD 1042.04 –USD 2298.94); Top 20%, >RM9619 (> USD 2298.94)

^#^ Bottom 40% (<RM 4,360 or < USD 1085.39), Middle 40% (RM 4360 to RM 9619 or USD 1085.39 –USD 2394.57), and Top 20% (>RM 9619 or > USD 2394.57)

Conversion rate, RM 1 = USD 0.249 [33]

**Table 5:** Association between levels of household income and prevalent odds of catastrophic health expenditure by a healthcare provider.

Characteristics	Ministry of Health’s Hospitals		Ministry of Education’s Hospitals	
	Adjusted Odds Ratio (95% CI) ^#^	P-Value	Adjusted Odds Ratio (95% CI) ^#^	P-Value
**Household Income** ^**1**^				
Bottom 40%	5.7 (1.3, 26.4)	**0.028**	0.8 (0.1, 5.0)	0.813
Middle 40%	5.5 (1.2, 25.1)	**0.023**	0.7 (0.1, 4.2)	0.689
Top 20%	1 (reference)		1 (reference)	

P-Heterogeneity = 0.3660

^1^ Bottom 40% (<RM 4,360 or < USD 1085.39), Middle 40% (RM 4360 to RM 9619 or USD 1085.39 –USD 2394.57), and Top 20% (>RM 9619 or > USD 2394.57)

^#^ Adjusted for age, age^2^, gender, ethnicity, geographical location, employment, household income and hospital clustering

Conversion rate, RM 1 = USD 0.249 [33]

The study was conducted following the World Medical Association Declaration of Helsinki. The proposal was reviewed and approved by the Medical Research and Ethics Committee of the Ministry of Health Malaysia, the University of Malaya Medical Centre, and Universiti Kebangsaan Malaysia, respectively. Written informed consent was obtained from participants before enrolment.

## Result

### Baseline characteristics

A total of 409 KTRs were recruited from 6 public health facilities in Klang Valley. However, only 398 participants provided their financial details in the survey. The participants’ socio-demographics and financing for post-transplantation care characteristics are tabulated in Table 1. The six public health facilities within Klang Valley accommodate 71% of KTR outside the region. The mean age of KTRs is at 47.2 years, with the majority being male patients at 56%. The KTRs are mainly employed at 63.1%, with tertiary education at 45.5% and married at 66.1%. Furthermore, 78.6% of the KTRs receive public financing for post-transplantation care. KTRs who perceived their financial burden on health expenditure to be extreme are at 12.8%.

Table 2 shows the medical characteristics of the KTRs by household income. Most of the KTRs underwent kidney transplantation from living donors (63.1%) in local transplant centres (60.8%). The KTRs also achieved a high compliance rate for outpatient follow-up at 84.4% and medication at 97.5%. MOH is the primary post-transplantation care provider, with 70.1% of the KTRs under their care.

### Factors associated with CHE

CHE was calculated using OOP payment for direct medical expenses that exceed 10% of household income. The number of KTRs who fall within this definition of catastrophic health expenditure is 93 KTRs (23.6%). The association of socioeconomic status and healthcare provider with CHE is shown in Table 3. Household income (p-value = 0.005) and the healthcare provider (p-value <0.001) are the only characteristics that are associated with CHE. Most of the KTRs employed (62.4%) and under the care of MOE hospitals (49.6%) suffered from CHE. The overall household income and healthcare providers are associated with CHE.

### Socioeconomic status and CHE

The crude and adjusted analysis for the association between socioeconomic status and CHE is shown in Table 4. The adjusted analysis shows that the bottom 40% of household income groups (OR 2.8, 95% CI 1.4 to 6.0) and Middle 40% of household income groups (OR 3.1, 95% CI 1.5 to 6.3) were more likely to incur CHE than those Top 20% household income groups. After adjusting for confounders, the KTRs under the care of MOE were associated with CHE (OR 8.6, 95% CI 5.0 to 14.9) compared to MOH hospitals.

### Healthcare provider and CHE

Table 5 reports the adjusted analysis for the association between levels of household income and prevalent odds of catastrophic health expenditure by the healthcare provider. The KTRs in the Bottom 40% (OR 5.7, 95% CI 1.3 to 26.4) and Middle 40% (OR 5.5, 95% CI 1.2 to 25.1) household income groups under the care of MOH hospitals were more prone to suffer from CHE.

## Discussion

The study illustrates the burden of post-transplantation care on KTRs attending outpatient clinics at six public tertiary healthcare facilities in the Klang Valley. To the best of the Authors’ knowledge, previous studies conducted on the financial burden of KT were limited to the healthcare system rather than to KTRs [16, 34, 40]. Conversely, our study is the first significant study conducted in a low and middle-income country examining the association of SES of KTRs on their post-transplantation catastrophic health expenditure (CHE).

The study of CHE is vital to measure equity in healthcare financing as well as the effectiveness of financial protection in achieving UHC. The findings provide insight into the determinants of CHE amongst KTRs. It demonstrated that more than one-fifth (23.6%) of the KTRs who primarily reside in the Klang Valley spent over 10% of their household income on post-transplantation care (direct medical expenditure), which was used as a CHE threshold in some previous studies [41–45]. This amount of spending on healthcare may reduce household expenditure on essential items such as food items, clothing, and housing, which may affect the family’s quality of life [46]. It was demonstrated by a study conducted in India that more than half of the KTRs’ family members are affected by post-transplantation care expenses [47].

All the participants (n = 409) in the study survey were recruited from six public hospitals in the Klang Valley, primarily hospitals managed by the MOH and supplemented by university hospitals operated by the MOE_. The Ministry of Finance funds public hospitals under MOH operation via the Consolidated Revenue Fund [48]. However, the hospitals by MOE were corporatised but non-privatised, prioritising public interest over the pure pursuit of profit [49]. As a result, the subsidies for hospital fees were significantly reduced, resulting in patients (excluding pensioners or government servants) paying more than the hospitals by MOH of RM 5 (~USD 1.24) [33] for each outpatient follow-up appointment, which includes laboratory investigations and medication. Despite the corporatisation of MOE hospitals, healthcare costs are relatively lower compared to private hospitals, even though KTRs have to pay OOP for consultation, laboratory investigations and medication [50, 51].

One of the most striking findings from the study is that educational attainment does not affect the CHE of post-transplantation care of KTRs. There was no statistically significant increment in CHE’s adjusted ratio in the educational attainment subgroup. Traditionally, higher education attainment was protective against CHE among cancer patients because they were considered savvier in procuring health services for a given level of health expenditure [52]. Although there are studies from Korea [53] and Türkiye [54] that showed CHE is associated with education level, the finding from this study supports what has been reported by published studies that showed no significant difference in educational attainment with CHE [55]. While the influence of lower SES often faces a higher likelihood of poor health outcomes because they lack the financial capability to pay for appropriate and adequate healthcare services [56]. The KTRs were asked how they cope with the OOP expenditure on their regular outpatient follow-up appointments and immunosuppressive therapy, with significant numbers of KTRs resorting to using their current household income and savings.

The study illustrated that CHE rates were significantly higher for KTRs under the MOE’s care at 49.6% compared to MOH’s care at 12.3%. Further analysis demonstrated that CHE was 8.6 times more prevalent for KTRs under the MOE’s care after adjusting for confounding. This indicates that the post-transplantation care at MOE hospitals would have a higher OOP payment than in MOH hospitals. Malaysian studies conducted on colorectal cancer patients [27] and gynaecological cancer patients [57] support our findings, which showed that long-term care in MOE hospitals would inevitably push half of these patients into CHE due to higher OOP payments compared to their ability to pay. It would be good to analyse the long-term effects of the corporatisation of hospitals by MOE on health equity, which is beyond the scope of this paper. However, we strongly recommend that the medical social workers in the hospital be engaged and work with non-governmental organisations (for example, the National Kidney Foundation) to assist these KTRs with financial difficulties to ease their burden in receiving long-term care.

Xu et al. reported that CHE is common in several low-income countries, countries in transition and middle-income countries [28]. This negative impact of health systems on households that can lead to impoverishment has long been ignored on the health policy agenda. CHE is not a new problem, although it may worsen because Malaysia’s public healthcare service use has been expanding rapidly, with an increase of 15% in the utilisation rate of public healthcare facilities [58]. This could be explained by a study by Sukeri et al. that revealed ischemic heart disease patients would still be required to pay OOP payment to a certain extent at private healthcare facilities in Malaysia even though they own health insurance [59]. The high OOP payment incurred at private healthcare facilities could explain why the Middle 40% income category seeks cheaper post-transplantation care at public healthcare facilities.

The study demonstrated a considerable increment of 3.1 times and 2.8 times for CHE for the Middle 40% household income and Bottom 40% household income category, respectively, compared to the Top 20% household income category. This study highlights the burden of OOP payments on lower-income and middle-income families. From the study findings, almost 10% of the KTRs seek loans from institutions and individuals as a coping strategy for the financial constraints of post-transplantation care. Similarly, Kruk et al. conducted a study in LMIC that indicated that 26% of households pay their healthcare expenses by receiving loans or selling their assets [60]. The repeated loan will disrupt the standard of living for the KTRs and their family members, either temporarily or in the long term. The long-term repercussion of this practice may lead to the KRTs being in debt [61] or pushed into poverty [47].

Based on the analysis of the study, the incidence of CHE is reported to be 5.7 times and 5.5 times higher in the Bottom 40% and Middle 40% household income groups, respectively, compared to the higher income group under the MOH’s care. While for MOE hospitals, both the Bottom 40% and Middle 40% of household income groups did not show statistical significance compared to the Top 20% household income groups. It is noteworthy that KTRs under MOH care suffer from CHE, mainly because of the implementation to restrict the supply of immunosuppressive medications to KTRs who underwent transplants overseas since 2011. Hence, these KTRs must resort to OOP payment to purchase the immunosuppressive drugs required [62]. Besides that, KTRs who underwent kidney transplantation at local private health facilities and transferred to MOH hospitals for post-transplantation care would need to pay OOP for immunosuppressive medications, which may lead to financial hardship. One way to reduce the financial stress of the KTRs in the Bottom 40% and Middle 40% household income groups is to provide targeted reimbursement for their immunosuppressive medications when needed. For example, in Hungary, reimbursement rates of 50%, 70%, 90% (for less severe chronic conditions) or 100% (for more severe, life-threatening diseases) are provided for medications that medical specialists prescribe for diseases considered to be more severe or longer lasting [63].

Generally, all KTRs can access healthcare services at government facilities. However, healthcare costs may financially burden the KTRs with lower incomes, even when public healthcare services are subsidised in Malaysia. Previous studies conducted in developing countries like China, Indonesia and India have shown that government subsidies in healthcare benefited patients with higher incomes rather than patients with lower incomes [29, 64]. Therefore, lower-income households were more likely to suffer CHE at any level than wealthier households [65]. The study affirms this conclusion by demonstrating that KTRs with lower household incomes have a higher risk of incurring catastrophic expenditures than higher household incomes.

The findings from our study echo the problem of high OOP and CHE that is not limited to the Bottom 40% but also the Middle 40% income group of KRTs in Malaysia. This was substantiated by a study conducted by Ng using data from the Department of Statistics that showed middle-income households were more likely to suffer from CHE compared to lower-income households [66]. There is a need to ensure that these patients are protected against CHE when seeking post-transplantation care. Despite Malaysia’s ability to provide equally affordable and accessible health services to the rich and poor with limited public funding, there should not be a trade-off in health outcomes with the ability to pay. The issue of CHE will not be solved by increasing household income. There is a need to change healthcare financing policies to effectively pool healthcare expenditure’s financial risk and narrow the socioeconomic inequity gap.

### Limitation of study

Our sample size was limited to public healthcare facilities in Klang Valley only because the number of KTRs in private healthcare facilities is few and scattered. It may not represent the Malaysian population and limit its generalizability to other settings. However, nearly half the KTRs population in Malaysia receive post-transplantation care at Klang Valley. The study was a cross-sectional design, which may not reflect the true CHE of post-transplantation care. The data collection may have recall bias on household income and expenditure. The recall period was limited to the preceding month post-transplantation to minimise bias. Some individuals with more inferior health status may refuse participation leading to some element of non-response bias. However, some studies have demonstrated that participants reported better outcomes than non-participants [67, 68]. It must be noted that the attending nephrologists were not engaged in confirming the compliance of KTRs for outpatient follow-up and medication.

### Strength of study

This multi-centre cross-sectional study is the first to investigate the financial burden of KTRs in Malaysia using the CHE approach. It achieved a statistical power representative of the Klang Valley and attained a response rate of 75%. The first author performed the data collection, ensuring that all participants’ instruction to administer the survey was standardised.

### The implication of study on policy

Malaysia has made significant progress towards achieving UHC, reflected in its impressive health indicators and accessibility of healthcare services. However, despite the low incidence of CHE, OOP payments remain a significant challenge for many Malaysians. In particular, the case of KTRs who face significant financial risks due to the long-term cost of their treatment. The findings act as a wake-up call for policymakers and clinicians to consider CHE burden of post-transplantation care instead of concentrating only on CHE of dialysis in Malaysia. Despite the government’s subsidies at public healthcare facilities, this study demonstrated that many KTRs experienced CHE, especially KTRs under the MOE hospitals’ care and those with lower income cared for by MOH hospitals.

Although Malaysia’s current definition of UHC focuses on ensuring access to essential health services and protection against financial risk for all, it does not fully capture the CHE faced by socioeconomically disadvantaged patients with chronic conditions such as the KTRs. This study provides significant evidence for the need to expand support programmes such as providing consultation with medical social workers working in partnership with civil society and reimbursement of immunosuppressive drugs in the public healthcare system for the vulnerable population, especially for KTRs in the bottom 40% and middle 40% household income groups. This study highlights the financial insecurity of post-transplantation care and the health inequity in the public healthcare system for other LMICs with a similar structure and socioeconomic composition.

## Conclusion

Malaysia provides UHC with near-free comprehensive health services through its public healthcare facilities [69]. However, the equal distribution of healthcare may only sometimes address the health inequity arising from KTRs’ socioeconomic backgrounds. This study demonstrated that KTRs and their family members in the Middle 40% and Bottom 40% income groups were 2.8 times and 3.1 times more likely to experience CHE than the Top 20% income group. Furthermore, KTRs in the MOE hospitals were 8.6 times more likely to suffer CHE compared to MOH hospitals. After adjusting for hospitals clustering, the KTRs in the Bottom 40% and Middle 40% showed a statistical significance for CHE. Given the financial insecurity of the sandwiched KTRs in the Middle 40%, they are more likely to face barriers or impoverishment seeking the needed health services. Granted that the national healthcare financing system focuses on access to health services, it must reexamine the policy to protect vulnerable households from financial catastrophe by minimising direct OOP healthcare expenditure. A revision of the healthcare policy is necessary to ensure UHC is equitable for all KTRs attending all public healthcare facilities, including MOH and MOE hospitals. By addressing these challenges, Malaysia can continue to make progress towards achieving UHC for all its people.

## Supporting information

S1 File Data set. (XLSX)

S2 File Questionnaire. (DOCX)

S3 File Validation of questionnaire. (DOCX)

## References

[1] Wagstaff A., et al., Progress on catastrophic health spending in 133 countries: a retrospective observational study. The Lancet Global Health, 2018. 6(2): p. e169–e179. doi: 10.1016/S2214-109X(17)30429-1 29248367

[2] Wang H., Torres L.V., and Travis P., Financial protection analysis in eight countries in the WHO South-East Asia Region. Bulletin of the World Health Organization, 2018. 96(9): p. 610. doi: 10.2471/BLT.18.209858 30262942 PMC6154066

[3] Xu K., et al., Household catastrophic health expenditure: a multicountry analysis. The lancet, 2003. 362(9378): p. 111–117. doi: 10.1016/S0140-6736(03)13861-5 12867110

[4] Gottret P.E. and Schieber G., Health financing revisited: a practitioner’s guide. 2006: World Bank Publications.

[5] World Health Organization. World Health Day 2018 –Lessons from Malaysia on Universal Health Coverage. 2018 02/12/2021]; Available from: https://www.who.int/malaysia/news/detail/18-04-2018-world-health-day-2018-%E2%80%93-lessons-from-malaysia-on-universal-health-coverage.

[6] Berman P., Ahuja R., and Bhandari L., The impoverishing effect of healthcare payments in India: new methodology and findings. Economic and Political Weekly, 2010: p. 65–71.

[7] Alam K. and Mahal A., Economic impacts of health shocks on households in low and middle income countries: a review of the literature. Globalization and health, 2014. 10(1): p. 1–18. doi: 10.1186/1744-8603-10-21 24708831 PMC4108100

[8] Wagstaff A. and E.v. Doorslaer, Catastrophe and impoverishment in paying for health care: with applications to Vietnam 1993–1998. Health economics, 2003. 12(11): p. 921–933. 14601155 10.1002/hec.776

[9] Kavosi Z., et al., Inequality in household catastrophic health care expenditure in a low-income society of Iran. Health policy and planning, 2012. 27(7): p. 613–623. doi: 10.1093/heapol/czs001 22279081

[10] Savedoff W.D. and Smith A.L.J.W., DC: Results for Development Institute, Achieving universal health coverage: learning from Chile, Japan, Malaysia and Sweden. 2011.

[11] Lo, J.Y.-R. and P. Allotey. World Health Day 2018 –Lessons from Malaysia on Universal Health Coverage. 2018 01/03/2021]; Available from: https://www.who.int/malaysia/news/detail/18-04-2018-world-health-day-2018-%E2%80%93-lessons-from-malaysia-on-universal-health-coverage.

[12] Gan Kim Soon P., et al., A qualitative examination of barriers and solutions to renal transplantation in Malaysia: Key-informants’ perspective. Plos one, 2019. 14(8): p. e0220411. doi: 10.1371/journal.pone.0220411 31404075 PMC6690507

[13] Axelrod D.A., et al., The interplay of socioeconomic status, distance to center, and interdonor service area travel on kidney transplant access and outcomes. Clinical Journal of the American Society of Nephrology, 2010: p. CJN. doi: 10.2215/CJN.04940610 . 20798250 PMC2994090

[14] Hod T. and Goldfarb-Rumyantzev A.S., The role of disparities and socioeconomic factors in access to kidney transplantation and its outcome. Renal failure, 2014. 36(8): p. 1193–1199. doi: 10.3109/0886022X.2014.934179 24988495

[15] Malaysia M.o.H., Malaysia National Health Accounts (MNHA). 2016, Planning & Development Division: Kuala Lumpur.

[16] Bavanandan S., et al., The Cost and Utility of Renal Transplantation in Malaysia. Transplantation Direct, 2015. 1(10): p. e45. doi: 10.1097/TXD.0000000000000553 27500211 PMC4946449

[17] Jensen C.E., Sørensen P., and Petersen K.D.J.D.M.J., In Denmark kidney transplantation is more cost-effective than dialysis. 2014. 61(3): p. A4796. 24814915

[18] Fu R., et al., Cost-effectiveness of deceased-donor renal transplant versus dialysis to treat end-stage renal disease: a systematic review. 2020. 6(2). 10.1097/TXD.0000000000000974 PMC7004633 32095508

[19] Abecassis, M., et al., Kidney transplantation as primary therapy for end-stage renal disease: a National Kidney Foundation/Kidney Disease Outcomes Quality Initiative (NKF/KDOQI™) conference. 2008. 3(2): p. 471–480. 10.2215/CJN.05021107 PMC2390948 18256371

[20] Andre M., et al., The UNOS renal transplant registry: Review of the last decade. 2014: p. 1–12. 26281122

[21] HS W. and BL G., 24th Report of the Malaysian Dialysis and Transplant Registry 2016. 2020.

[22] Tonelli M., et al., Systematic review: kidney transplantation compared with dialysis in clinically relevant outcomes. 2011. 11(10): p. 2093–2109. 10.1111/j.1600-6143.2011.03686.x 21883901

[23] Vos T., et al., Global, regional, and national incidence, prevalence, and years lived with disability for 301 acute and chronic diseases and injuries in 188 countries, 1990–2013: a systematic analysis for the Global Burden of Disease Study 2013. 2015. 386(9995): p. 743–800. 10.1016/S0140-6736(15)60692-4 PMC4561509 26063472

[24] Hajian-Tilaki K., Sample size estimation in epidemiologic studies. Caspian journal of internal medicine, 2011. 2(4): p. 289. 24551434 PMC3895825

[25] Fincham J.E., Response rates and responsiveness for surveys, standards, and the Journal. American journal of pharmaceutical education, 2008. 72(2). doi: 10.5688/aj720243 18483608 PMC2384218

[26] Seng W.H. and Leong G.B., 23rd Report of the Malaysian Dialysis and Transplant Registry 2015. 2016, Malaysian Society of Nephrology: Kuala Lumpur.

[27] Azzani M., et al., Catastrophic health expenditure among colorectal cancer patients and families: a case of Malaysia. Asia Pacific Journal of Public Health, 2017. 29(6): p. 485–494. doi: 10.1177/1010539517732224 29019257

[28] Xu K. and World Health Organization, Distribution of health payments and catastrophic expenditures methodology. 2005.

[29] O’Donnell O., et al., The incidence of public spending on healthcare: comparative evidence from Asia. The World Bank Economic Review, 2007. 21(1): p. 93–123.

[30] Xu K., et al., Exploring the thresholds of health expenditure for protection against financial risk. World health report, 2010. 3: p. 328–33.

[31] Choi J.W., et al., Catastrophic health expenditure according to employment status in South Korea: a population-based panel study. BMJ open, 2016. 6(7): p. e011747. doi: 10.1136/bmjopen-2016-011747 27456329 PMC4964244

[32] Department of Statistics Malaysia. Report of Household Income And Basic Amenities Survey 2016. 2017 20/08/2018]; Available from: https://dosm.gov.my/v1/index.php?r=column/cthemeByCat&cat=120&bul_id=RUZ5REwveU1ra1hGL21JWVlPRmU2Zz09&menu_id=amVoWU54UTl0a21NWmdhMjFMMWcyZz09.

[33] Bank Negara Malaysia. Exchange Rates. 2020 01/06/2021]; Available from: https://www.bnm.gov.my/exchange-rates?p_p_id=bnm_exchange_rate_display_portlet&p_p_lifecycle=0&p_p_state=normal&p_p_mode=view&_bnm_exchange_rate_display_portlet_monthStart=11&_bnm_exchange_rate_display_portlet_yearStart=2020&_bnm_exchange_rate_display_portlet_monthEnd=11&_bnm_exchange_rate_display_portlet_yearEnd=2020&_bnm_exchange_rate_display_portlet_sessionTime=1700&_bnm_exchange_rate_display_portlet_rateType=MR&_bnm_exchange_rate_display_portlet_quotation=rm.

[34] Salamzadeh J., et al., Costs of treatment after renal transplantation: is it worth to pay more? 2014. 13(1): p. 271. PMC3985242 24734080

[35] Greene W., Econometric Analysis. New Jersey: PrenticeHall International. 1997, Inc.

[36] Hosmer D.W. Jr, Lemeshow S., and Sturdivant R.X., Applied logistic regression. Vol. 398. 2013: John Wiley & Sons.

[37] Rabe-Hesketh S. and Skrondal A., Multilevel and longitudinal modeling using Stata. 2008: STATA press.

[38] Kleinbaum D.G. and Klein M., Introduction to logistic regression, in Logistic regression. 2010, Springer. p. 1–39.

[39] Schünemann H., et al., The GRADE approach and Bradford Hill’s criteria for causation. Journal of Epidemiology & Community Health, 2011. 65(5): p. 392–395. doi: 10.1136/jech.2010.119933 20947872

[40] Eriksson J.K., et al., Healthcare costs in chronic kidney disease and renal replacement therapy: a population-based cohort study in Sweden. BMJ open, 2016. 6(10): p. e012062. doi: 10.1136/bmjopen-2016-012062 27855091 PMC5073563

[41] O’donnell O., et al., Analyzing health equity using household survey data. Washington, DC: World Bank, 2008.

[42] Arsenijevic J., et al., Catastrophic health care expenditure among older people with chronic diseases in 15 European countries. PLoS One, 2016. 11(7): p. e0157765. doi: 10.1371/journal.pone.0157765 27379926 PMC4933384

[43] Limwattananon S., Tangcharoensathien V., and Prakongsai P., Catastrophic and poverty impacts of health payments: results from national household surveys in Thailand. Bulletin of the World Health Organization, 2007. 85: p. 600–606. doi: 10.2471/blt.06.033720 17768518 PMC2636377

[44] Raban M.Z., Dandona R., and Dandona L., Variations in catastrophic health expenditure estimates from household surveys in India. Bulletin of the World Health Organization, 2013. 91: p. 726–735. doi: 10.2471/BLT.12.113100 24115796 PMC3791647

[45] Loganathan T., et al., Household catastrophic healthcare expenditure and impoverishment due to rotavirus gastroenteritis requiring hospitalization in Malaysia. PLoS One, 2015. 10(5): p. e0125878. doi: 10.1371/journal.pone.0125878 25941805 PMC4420491

[46] Kawabata K., Xu K., and Carrin G., Preventing impoverishment through protection against catastrophic health expenditure. 2002, SciELO Public Health. PMC2567587 12219150

[47] Ramachandran R. and Jha V., Kidney transplantation is associated with catastrophic out of pocket expenditure in India. PLoS One, 2013. 8(7): p. e67812. doi: 10.1371/journal.pone.0067812 23861812 PMC3701634

[48] Kananatu K., Healthcare financing in Malaysia. Asia Pacific Journal of Public Health, 2002. 14(1): p. 23–28. 12597514 10.1177/101053950201400106

[49] Virk A., et al., Hybrid Organizations in Health Systems: The Corporatization of Malaysia’s National Heart Institute. Health Systems & Reform, 2020. 6(1): p. e1833639. doi: 10.1080/23288604.2020.1833639 33314988

[50] Quek D. The Malaysian healthcare system: a review. in Intensive workshop on health systems in transition: 29–30 April 2009; Kuala Lumpur. 2009.

[51] Thomas S., Beh L., and Nordin R.B., Health care delivery in Malaysia: changes, challenges and champions. Journal of public health in Africa, 2011. 2(2). 10.4081/jphia.2011.e23 PMC5345496 28299064

[52] Doshmangir L., et al., Incidence of Catastrophic Health Expenditure and Its Determinants in Cancer Patients: A Systematic Review and Meta-analysis. Applied Health Economics and Health Policy, 2021. 19(6): p. 839–855. doi: 10.1007/s40258-021-00672-2 34318445

[53] Lee M. and Yoon K., Catastrophic health expenditures and its inequality in households with cancer patients: A panel study. Processes, 2019. 7(1): p. 39.

[54] Yardim M.S., Cilingiroglu N., and Yardim N., Catastrophic health expenditure and impoverishment in Turkey. Health policy, 2010. 94(1): p. 26–33. doi: 10.1016/j.healthpol.2009.08.006 19735960

[55] Choi J.-W., et al., Association between chronic disease and catastrophic health expenditure in Korea. BMC health services research, 2015. 15(1): p. 26. doi: 10.1186/s12913-014-0675-1 25608983 PMC4307618

[56] Li C., Young B.-R., and Jian W., Association of socioeconomic status with financial burden of disease among elderly patients with cardiovascular disease: evidence from the China Health and Retirement Longitudinal Survey. BMJ open, 2018. 8(3): p. e018703. doi: 10.1136/bmjopen-2017-018703 29567841 PMC5875679

[57] Liew C.H., Shabaruddin F.H., and Dahlui M.. The Burden of Out-of-Pocket Expenditure Related to Gynaecological Cancer in Malaysia. in Healthcare. 2022. MDPI. doi: 10.3390/healthcare10102099 36292545 PMC9601824

[58] Malaysia M.o.H., National Health and Morbidity Survey 2015 (NHMS 2015). Volume III–Healthcare Demand. 2015, Institute for Public Health Kuala Lumpur.

[59] Sukeri S., Mirzaei M., and Jan S., Does tax-based health financing offer protection from financial catastrophe? Findings from a household economic impact survey of ischaemic heart disease in Malaysia. International health, 2017. 9(1): p. 29–35. doi: 10.1093/inthealth/ihw054 28028130

[60] Kruk M.E., Goldmann E., and Galea S., Borrowing and selling to pay for health care in low-and middle-income countries. Health Affairs, 2009. 28(4): p. 1056–1066. doi: 10.1377/hlthaff.28.4.1056 19597204

[61] Van Doorslaer E., et al., Catastrophic payments for health care in Asia. Health economics, 2007. 16(11): p. 1159–1184. doi: 10.1002/hec.1209 17311356

[62] Director-General of Health Malaysia. Surat Pekeliling Ketua Pengarah Kesihatan Bil. 12/2011—Dasar Bekalan Ubat Immunosuppressant Kepasa Pesakit Yang Telah Menjalani Pembedahan Transplan Organ. 2011 [cited 2018 06/06/2018]; Available from: http://www.moh.gov.my/index.php/database_stores/attach_download/312/327.

[63] Vogler S., et al., Medicines reimbursement policies in Europe. 2018.

[64] Gwatkin D.R., How much would poor people gain from faster progress towards the Millennium Development Goals for health? The Lancet, 2005. 365(9461): p. 813–817. doi: 10.1016/S0140-6736(05)17992-6 15733726

[65] Wagstaff A., Poverty and health sector inequalities. Bulletin of the world health organization, 2002. 80: p. 97–105. 11953787 PMC2567730

[66] Ng C.-W., Universal health coverage assessment: Malaysia. Global Network for Health Equity (GNHE), 2015.

[67] Berg N., Non-response bias. Encyclopedia of social measurement 2: 865–873. Kempf-Leonard K., ed. 2010, London: Academic Press.

[68] Cheung K.L., et al., The impact of non-response bias due to sampling in public health studies: a comparison of voluntary versus mandatory recruitment in a Dutch national survey on adolescent health. BMC public health, 2017. 17(1): p. 276. doi: 10.1186/s12889-017-4189-8 28330465 PMC5363011

[69] Ng C.W., et al., Universal health coverage in Malaysia: issues and challenges. Revisiting Malaysia’s Population–Development Nexus, 2014: p. 175.

