# Supplementary material for: Effect of socioeconomic status and healthcare provider on post-transplantation care in Malaysia: A multi-centre survey of kidney transplant recipients
Source: PLoS One. 2023 Apr 19;18(4):e0284607. doi: 10.1371/journal.pone.0284607 (PMC10115286; doi:10.1371/journal.pone.0284607)
Supplement: S2 File — (DOCX) [file pone.0284607.s002.docx]

**S2 File: Questionnaire**

Tick (X) or fill in details for each question as indicated.

| Q1 | Were you **physically** **prepared** for the kidney transplant? | \| Yes \|  \| No \|  \| \| --- \| --- \| --- \| --- \| \|  \|  \| | | | |
| --- | --- | --- | --- | --- | --- | --- | --- | --- | --- | --- | --- |
| Q2 | Were you **emotionally** **prepared** for the kidney transplant? | \| Yes \|  \| No \|  \| \| --- \| --- \| --- \| --- \| \|  \|  \| | | | |
| Q3 | Were you **spiritually** **prepared** for the kidney transplant? | \| Yes \|  \| No \|  \| \| --- \| --- \| --- \| --- \| \|  \|  \| | | | |
| Q4 | Were you **financially** **prepared** for the kidney transplant? | \| Yes \|  \| No \|  \| \| --- \| --- \| --- \| --- \| \|  \|  \| | | | |
| Q5 | Do you go for your follow-up **appointment regularly**? | \| Yes \|  \| No \|  \| \| --- \| --- \| --- \| --- \| \|  \|  \| | | | |
| Q6 | Do you regularly take your **medication as prescribed**? | \| Yes \|  \| No \|  \| \| --- \| --- \| --- \| --- \| \|  \|  \| | | | |
| Q7 | Estimated **monthly** **household income** - total income from **personal and family income** (includes wages, salary, bonus, self-employment income, pension, retirement fund, interest, investment and rent) | RM……….…………………………………………………………………............ | | | |
| Q8 | How was payment for **medical expenses** obtained (you may choose more than one option) | \| Household **income** \|  \| **Saving** \|  \| \| --- \| --- \| --- \| --- \| \|  \|  \| \| Bank loan \|  \| Loan from **relatives/friends** \|  \| \|  \|  \| \| **Selling assets** (e.g. furniture, jewellery, car, appliances) \|  \| **Reducing** household **spending** \|  \| \|  \|  \| \| **Private** company (Staff or family member of staff of the company) \|  \| **Government**  (Government servant or family member of a government servant) \|  \| \|  \|  \| \| Employee Provident Fund (**EPF**) \|  \| Non-governmental organizations (**NGO**) \|  \| \|  \|  \| \| Payment or reimbursement of **health insurance** plan \|  \| Community welfare department \|  \| \|  \|  \| \| Others, please mention  **……………………..** \|  \|  \|  \| \|  \| | | | |
| Q9 | **In the last 4 weeks**, what is the estimated **expenditure** that your **household** spends on? | **TOTAL** monthly expenditure (you and your family) | | | RM |
|  |  | **Food** (include the value of any food that was produced and consumed by the household, and exclude alcohol, tobacco and restaurant meals) | | | RM |
|  |  | **Housing** (include mortgage and rent) | | | RM |
|  |  | Cooking **gas**, **electricity** bill, **water** bill, **telephone** bill, **internet** bill | | | RM |
|  |  | **Education fees** and **school supplies** | | | RM |
|  |  | **Health care cost** (not including insurance payment) | | | RM |
|  |  | **Clothing** and **footwear** | | | RM |
|  |  | **Furnishings**, **household** equipment and routine household **maintenance** | | | RM |
|  |  | **Insurance** premiums or any prepaid health plans | | | RM |
|  |  | **Recreation** services and **culture** | | | RM |
|  |  | **Transportation** | | | RM |
|  |  | **Restaurants** and **hotels** | | | RM |
|  |  | All other **goods and services**  (**Goods** e.g. toy, CDs, accessories, electronic gadgets, computers)  (**Services** e.g. haircut, postal services, vehicle repair) | | | RM |
| Q10 | What is the **cost** for **one-way fare** for your last follow-up appointment? | RM……………………………………………………………………………….. | | | |
| Q11 | Number of follow-up **appointments** in **1 year** | \| 1 \|  \| 7 \|  \| \| --- \| --- \| --- \| --- \| \| 2 \|  \| 8 \|  \| \| 3 \|  \| 9 \|  \| \| 4 \|  \| 10 \|  \| \| 5 \|  \| 11 \|  \| \| 6 \|  \| 12 \|  \| | | | |
| Q12 | What is the **usual cost** of your **follow-up appointment**? (if unknown, state the cost for your last follow-up appointment) | Registration **fee** | | RM | |
|  |  | **Blood investigation** and imaging (e.g. x-ray, CT scan, MRI, ultrasound) | | RM | |
|  |  | **Medication** (for 1 month) | | RM | |
|  |  | Others (**food** and beverage) | | RM | |
|  |  | **Total amount** of **expenditure** (if specific breakdown unknown) | | RM | |
| Q13 | Do you use **vitamins, supplements, herbal or traditional remedies**? | \| Yes \|  \| No \|  \| \| --- \| --- \| --- \| --- \| \|  \|  \|   If yes**, in the** last 4 weeks **how much money was spent on these types of health-related items?**  RM………………………………. | | | |
| Q14 | In the **last 4 weeks**, were you **admitted** in the hospital? | \| Yes \|  \| No \|  \| \| --- \| --- \| --- \| --- \| \|  \|  \|   If no, please proceed to question 70 | | | |
| Q15 | Why were you admitted into the hospital? | ……………………………………………………………………………………… | | | |
| Q16 | What is the **cost** of your **hospital admission**? (if unknown, state the cost for your last follow-up appointment) | Registration **fee** | RM | | |
|  |  | **Bed charges** | RM | | |
|  |  | **Procedure charges** | RM | | |
|  |  | **Blood investigation** and **imaging** (e.g. x-ray, CT scan, MRI, ultrasound) | RM | | |
|  |  | **Medication** | RM | | |
|  |  | Others (**food** and beverage) | RM | | |
|  |  | **Total amount** of **expenditure** (if specific breakdown unknown) | RM | | |

Circle (O) as indicated. If you are unsure about how to answer a question, please give the best answer you can.

|  |  | Not at all | A little | A moderate amount | Very much | An extreme amount |
| --- | --- | --- | --- | --- | --- | --- |
| Q17 | What is the degree of **difficulty paying** for your **healthcare**? | **1** | **2** | **3** | **4** | **5** |
