# Supplementary material for: Effect of socioeconomic status and healthcare provider on post-transplantation care in Malaysia: A multi-centre survey of kidney transplant recipients
Source: PLoS One. 2023 Apr 19;18(4):e0284607. doi: 10.1371/journal.pone.0284607 (PMC10115286; doi:10.1371/journal.pone.0284607)
Supplement: S3 File — (DOCX) [file pone.0284607.s003.docx]

S3 File. Validation of Questionnaire

| **Item** | **Relevance** | | **Consistency** | | **Representativeness** | | **Clarity** | | **Comments** | **Results** |
| --- | --- | --- | --- | --- | --- | --- | --- | --- | --- | --- |
|  | **CVI** | **Kappa** | **CVI** | **Kappa** | **CVI** | **Kappa** | **CVI** | **Kappa** |  |  |
| Q1 | 1.000 | 1.000 | 1.000 | 1.000 | 1.000 | 1.000 | 1.000 | 1.000 | - | VALIDATED |
| Q2 | 1.000 | 1.000 | 1.000 | 1.000 | 1.000 | 1.000 | 1.000 | 1.000 | - | VALIDATED |
| Q3 | 1.000 | 1.000 | 1.000 | 1.000 | 1.000 | 1.000 | 1.000 | 1.000 | - | VALIDATED |
| Q4 | 1.000 | 1.000 | 1.000 | 1.000 | 1.000 | 1.000 | 1.000 | 1.000 | - | VALIDATED |
| Q5 | 1.000 | 1.000 | 1.000 | 1.000 | 1.000 | 1.000 | 1.000 | 1.000 | - | VALIDATED |
| Q6 | 1.000 | 1.000 | 1.000 | 1.000 | 1.000 | 1.000 | 1.000 | 1.000 | - | VALIDATED |
| Q7 | 1.000 | 1.000 | 1.000 | 1.000 | 1.000 | 1.000 | 1.000 | 1.000 | Remove categories of income and leave it open | VALIDATED |
| Q8 | 0.857 | 0.849 | 1.000 | 1.000 | 1.000 | 1.000 | 1.000 | 1.000 | Rephrase health financing. Provide “others” as option. | VALIDATED |
| Q9 | 1.000 | 1.000 | 1.000 | 1.000 | 1.000 | 1.000 | 1.000 | 1.000 | Change duration to 1 month instead of 1 week – difficult to estimate. Give examples of Goods and services | VALIDATED |
| Q10 | 1.000 | 1.000 | 1.000 | 1.000 | 1.000 | 1.000 | 1.000 | 1.000 | - | VALIDATED |
| Q11 | 1.000 | 1.000 | 1.000 | 1.000 | 1.000 | 1.000 | 1.000 | 1.000 | - | VALIDATED |
| Q12 | 1.000 | 1.000 | 1.000 | 1.000 | 1.000 | 1.000 | 1.000 | 1.000 | - | VALIDATED |
| Q13 | 1.000 | 1.000 | 1.000 | 1.000 | 1.000 | 1.000 | 1.000 | 1.000 | - | VALIDATED |
| Q14 | 1.000 | 1.000 | 1.000 | 1.000 | 1.000 | 1.000 | 1.000 | 1.000 | - | VALIDATED |
| Q15 | 1.000 | 1.000 | 1.000 | 1.000 | 1.000 | 1.000 | 1.000 | 1.000 | - | VALIDATED |
| Q16 | 0.857 | 0.849 | 1.000 | 1.000 | 1.000 | 1.000 | 1.000 | 1.000 | - | VALIDATED |
| Q17 | 1.000 | 1.000 | 1.000 | 1.000 | 1.000 | 1.000 | **0.714** | **0.658** | Use Likert scale | VALIDATED after revision |
